# Cervical Cancer Progression in Patients Waiting for Radiotherapy Treatment at a Referral Center in Ethiopia: A Longitudinal Study

**DOI:** 10.1200/GO.22.00435

**Published:** 2023-05-22

**Authors:** Jilcha D. Feyisa, Mathewos A. Woldegeorgis, Girum T. Zingeta, Kedir H. Abegaz, Yemane Berhane

**Affiliations:** ^1^ Department of Oncology, Saint Paul's Hospital Millennium Medical College, Addis Ababa, Ethiopia; ^2^ Department of Oncology, School of Medicine, Addis Ababa University, Addis Ababa, Ethiopia; ^3^ Department of Biostatistics & Health Informatics, Madda Walabu University, Robe, Ethiopia; ^4^ Department of Epidemiology and Biostatics, Addis Continental Institute of Public Health, Addis Ababa, Ethiopia

## Abstract

**PURPOSE:**

Nonmetastatic cervical cancer is curable and can be treated with radiotherapy (RT). A delay in receiving treatment because of long waiting times results in upstaging of the disease stage and negatively affects the treatment outcomes. However, real-world evidence that progression occurs while waiting for treatment is scarce in low-income countries. We evaluated the impact of long waiting times for RT in patients with cervical cancer at a referral center in Ethiopia.

**METHODS:**

A longitudinal study was conducted from January 5, 2019, to May 30, 2020, to address the objectives of this study. Patients with pathologically diagnosed cervical cancer with stage IIB to stage IVA were included in the study. We used Kaplan-Meier analysis to assess overall survival with time. Multivariate Cox regression analysis, using the backward likelihood ratio selection method, was used to fit the final model.

**RESULTS:**

The median waiting time for radical RT after diagnosis was 477 days. Waiting for more than 51 days for RT results in disease progression. Of the 115 patients included in this study, 59 (51.3%) died during the study period. A delay in waiting (adjusted hazard ratio, 3; 95% CI, 1.7 to 4.9) was significantly associated with disease progression and decreased survival.

**CONCLUSION:**

Waiting time to receive RT is very long. Urgent action is required to significantly reduce waiting times and improve the survival of patients with cervical cancer.

## INTRODUCTION

Cervical cancer is the second most frequent cancer among women in Ethiopia.^1-3^ In Ethiopia, 35.9 new cases of cervical cancer are diagnosed and 22.6 deaths per 100,000 women occur annually.^4^ According to the Addis Ababa Cancer Registry, cervical cancer accounts for 14.7% of all cancer cases.^1^

**CONTEXT**

**Key Objective**
What is the impact of a long wait for radiotherapy (RT) in low-income countries on the outcome for patients with cervical cancer?
**Knowledge Generated**
In Ethiopia, the median waiting time between diagnosis and receiving radical dose RT for patients with cervical cancer was 477 days. More than half of these patients died before receiving RT.
**Relevance**
It is essential to expand access to RT services in lower-income nations to reduce wait times and enhance patients' survival.

Nonmetastatic cervical cancer is a highly curable malignancy. Radiotherapy (RT) is the mainstay treatment for cervical cancer and plays a vital role in its management. Concurrent chemoradiotherapy (CCRT) has been established as the standard treatment for locally advanced cervical cancer.^5^

The initiation time is vital for cervical cancer treatment. The optimal time to initiate RT for cure is 6 weeks.^6,7^ Upstaging usually occurs after a delay of 45-60 days.^7^ There was a 15% decrease in 3-year overall survival (OS) for those who waited 40 days.^6^ The waiting time for the initiation of RT for cervical cancer varies worldwide.^6-8^ In Ontario, Canada, the median waiting time for patients with cervical cancer is 27.2 days from diagnosis to the commencement of RT, whereas it is 108 days in South Africa.^6,7^ Approximately 72.2% of patients with cervical cancer in Brazil receive RT within 41 days of diagnosis.^8^

RT treatment machines are rarely available in low- and middle-income countries.^9^ Ethiopia, a country with an estimated population of 120 million, had only one Cobalt-60 RT machine at Tikur Anbessa Specialized Hospital (TASH, Addis Ababa, Ethiopia), until it acquired its first linear accelerator very recently. This results in longer waiting times and overwhelming problems in patient treatment. However, real-world evidence of cancer progression because of treatment delay is scarce in low-income countries. A study showed a survival rate of patients with cervical cancer in Ethiopia who received any form of oncologic therapy, including surgery or RT.^10^ However, it did not assess the extent of waiting time for RT or the effect on the natural history of the disease. This study aimed to assess the extent of delay in receiving RT treatment and the effect of delay on the natural disease course of patients with cervical cancer at TASH, in Ethiopia.

## METHODS

A longitudinal study was conducted at TASH, Addis Ababa, Ethiopia, from January 5, 2019, to May 30, 2020. TASH is the only referral cancer treatment center with available RT services. For this reason, patients with cancer are referred to the center from all over the country.

The clinical condition of each patient at different time points, pre- and postradiation therapy, was evaluated during clinic visits and from their medical charts. When patients were allocated a start date for treatment, they were called on the phone by a member of the department. The status of patients who could not visit the hospital for treatment or follow-up was assessed via phone interviews with the patients or their caregivers.

Stage assignment was based on the 2018 revised staging system from the International Federation of Gynecology and Obstetrics.^10^ Clinical assessment and imaging modalities, including abdominopelvic ultrasound, pelvic magnetic resonance imaging (MRI), and plain chest radiography, were used for the staging purposes. Staging was performed by gynecologic oncologists, clinical oncologists, or clinical oncology resident physicians during patient evaluation.

The study included all individuals who were diagnosed with cervical cancer and booked for the radical intent of RT treatment in the TASH, within the specified duration of research undertaking. Inclusion criteria included individuals who were pathologically diagnosed with stage IIB to stage IVA, with a good performance status of Eastern Cooperative Oncology Group (ECOG) 0 to ECOG II, and had adequate clinical and laboratory documentation of the patient course. The exclusion criteria included patients who received palliative RT immediately during the first visit, regardless of stage and metastatic cervical cancer. On the basis of these criteria, the study was designed to reduce the possibility of bias and ensure the quality of data collection.

CCRT was administered for cervical cancer combining Cobalt-60 teletherapy with a high-dose-rate (HDR) brachytherapy boost. The Cobalt-60 teletherapy was administered up to a total of 46 Gy, delivered in 2 Gy fractions daily, whereas the HDR brachytherapy boost was given in four fractions of 6.5 Gy each once every week, to a total of 26 Gy. A conventional two-dimensional RT technique was used, and chemotherapy was administered concurrently using cisplatin 40 mg per meter square of the body surface area once every week.

A data extraction sheet was prepared on the basis of the objectives of this study and similar research experience in other countries. Two trained clinical oncology resident physicians at the TASH Oncology Department collected the data and made phone assessments. A pretest was conducted on 10 medical charts before data collection began.

Ethical clearance was obtained from the Addis Continental Institute of Public Health Institutional Review Board. Permission to review patient charts and contact patients via the phone was obtained from the oncology department. Written informed consent to participate in the study was obtained during the hospital visits. Confidentiality of the information was maintained throughout the study by excluding names as identification in the data extraction form and the data used only for the purpose of the conducted study.

After checking for completeness and consistency, the data were entered into Epi Info version 7.2.2.6 and analyzed using SPSS version 23. Kaplan-Meier analysis was used to assess OS with time.

## RESULTS

A total of 115 patients were included in this study. The mean age of the participants was 51 years with a standard deviation of 10. Their age ranged from 30 to 75 years. Squamous cell carcinoma was the leading histologic type 103 (89.6%), followed by adenocarcinoma 6 (5.2%). Physical examination performed at initial presentation revealed parametrial invasion in 113 (98.3%) patients (Table 1).

**TABLE 1:**
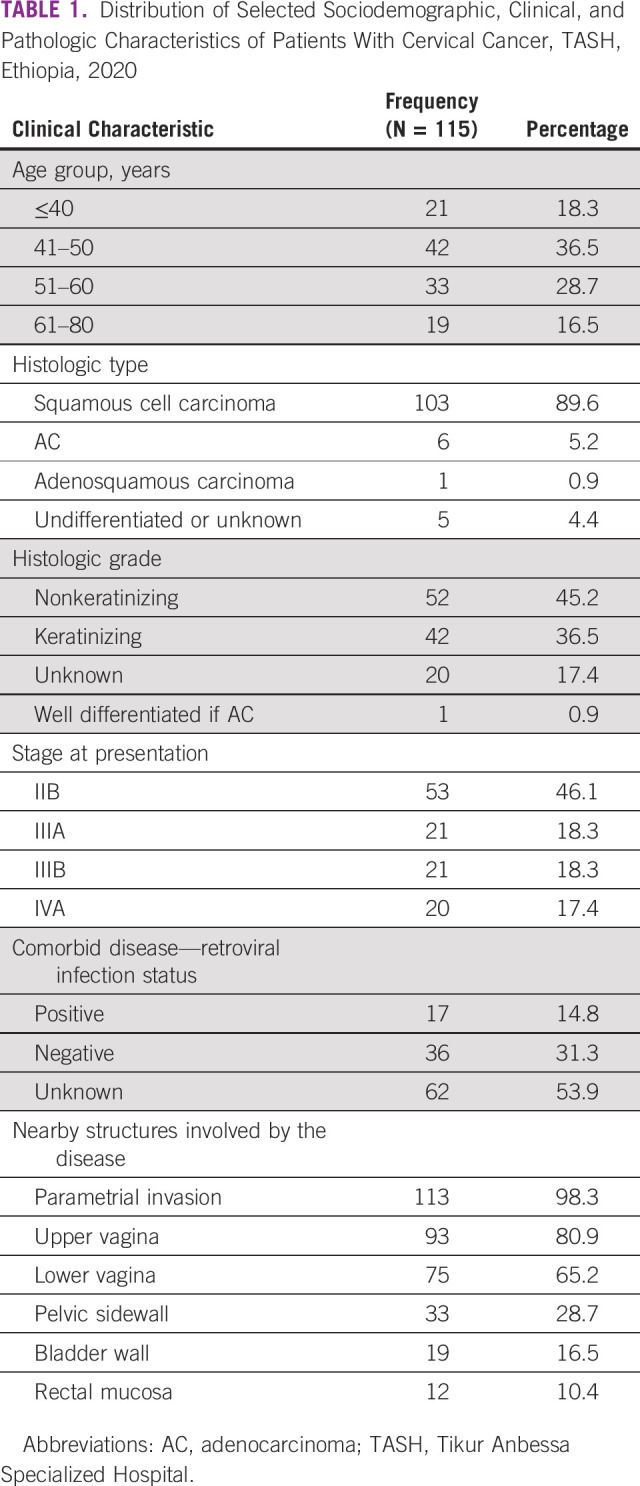
Distribution of Selected Sociodemographic, Clinical, and Pathologic Characteristics of Patients With Cervical Cancer, TASH, Ethiopia, 2020

The median time delay for booking after pathologic diagnosis was 19 days. The median waiting time from booking to the call for CCRT to the start of RT was 458 days. The total median time from diagnosis to treatment was 477 days. The median time to disease progression was 51 days (Table 2).

**TABLE 2:**
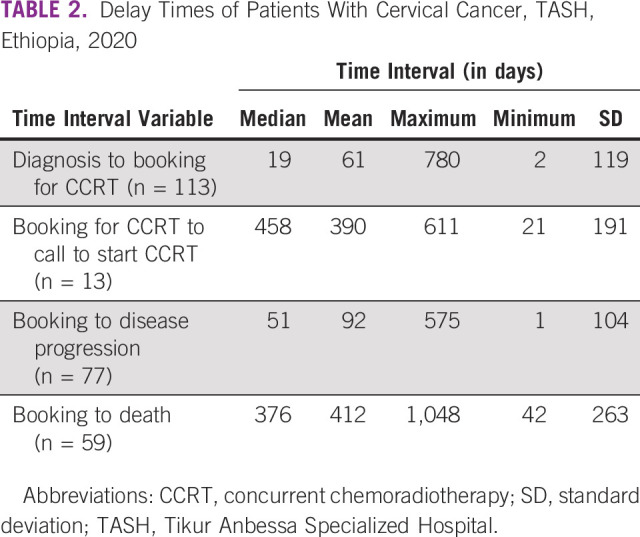
Delay Times of Patients With Cervical Cancer, TASH, Ethiopia, 2020

**FIG 1:**
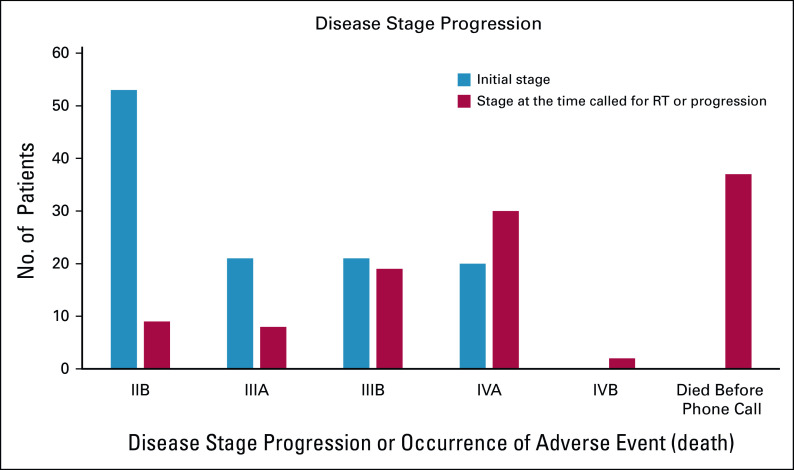
Stage at initial presentation and at the time called for RT treatment or during the progression of patients with cervical cancer, TASH, Ethiopia, 2020. RT, radiotherapy; TASH, Tikur Anbessa Specialized Hospital.

The stage at presentation was recorded for all 115 patients. Almost half of the patients were diagnosed with stage IIB disease (n = 53, 46.1%), whereas 21 (18.3%) presented with stage IIIA or IIIB disease. The remaining 20 (17.4%) patients were diagnosed with stage IVA. During the waiting period for RT, the stage or patient status was reassessed for 105 patients. Stage migration and occurrence of adverse events (death) were significant during the waiting period. Specifically, the number of patients in stage IIB decreased from 53 (46.1%) to 9 (7.8%), the number of patients in stage IIIA decreased from 21 (18.3%) to 8 (7%), the number of patients in stage IIIB decreased from 21 (18.3%) to 19 (16.5%), the number of patients in stage IVA increased from 20 (17.4%) to 30 (26.1%), and 2 (1.8%) patients developed distant metastasis to the lungs (stage IVB). Furthermore, 37 patients died before receiving a phone call for RT (Fig 1).

**FIG 2:**
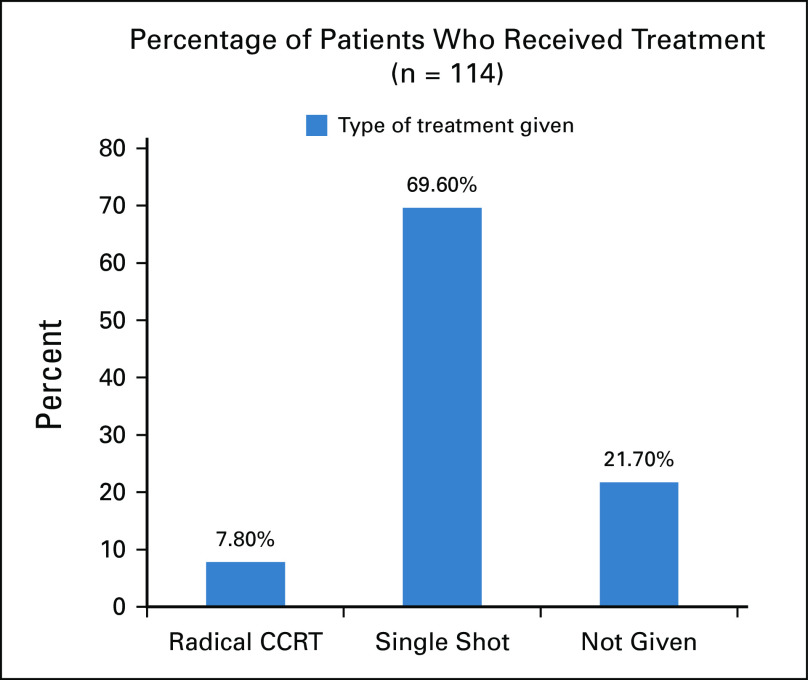
Radiation therapy regimen received by patients with cervical cancer, TASH, Ethiopia, 2020. CCRT, concurrent chemoradiotherapy; TASH, Tikur Anbessa Specialized Hospital.

Of the 115 patients booked to receive radical dose of CCRT, 69.9% (n = 80) ultimately received a single shot of palliative RT because of disease progression. Only 7.8% of patients (n = 9) received radical CCRT with HDR brachytherapy boost, and 21.7% of patients (n = 25) did not receive RT at all (Fig 2).

**FIG 3:**
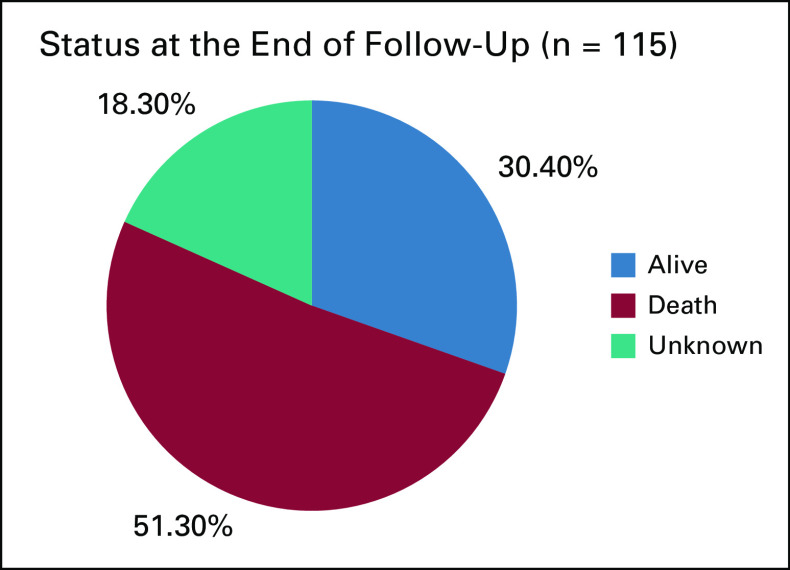
Status of patients with cervical cancer at the end of follow-up, TASH, Ethiopia, 2020. TASH, Tikur Anbessa Specialized Hospital.

Fifty-nine (51.30%) of them were found to be dead during the study period. Thirty-five (30.4%) patients were alive at the time of analysis. The status of 21 patients (18.3%) was unknown (Fig 3).

**FIG 4:**
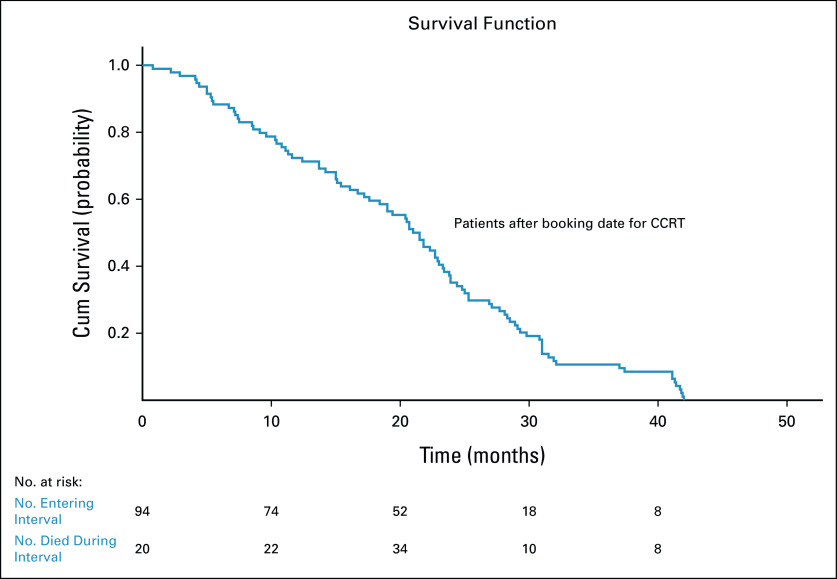
Kaplan-Meier plot of the survival function for patients with cervical cancer after booking date for CCRT, TASH, Ethiopia, 2020. CCRT, concurrent chemoradiotherapy; TASH, Tikur Anbessa Specialized Hospital.

The mean and median survival times were 20.1 months (95% CI, 18.3 to 22.7) and 21 months (95% CI, 18.3 to 23.8), respectively. The OS rate showed a decline starting from the early days, as shown by Kaplan-Meier curves (Fig 4).

Twenty-one variables were selected for the bivariable analysis, among which waiting time, stage at presentation, distant metastases during the waiting time, hydronephrosis during the waiting time, and type of treatment were significantly associated with survival and were selected for multivariate Cox regression analysis with a *P* value of <.25 for 115 patients.

The final model was established for the 87 patients with cervical cancer. The final model was significant (*X*^2^ (9), 94.376; *P* = .000). The stage at presentation, mucosal involvement, waiting time, and type of RT treatment were variables that explained the model. Patients with stage IIIB were 2.2 times more likely to die than those with stage IIB disease (adjusted hazard ratio [AHR] and 95% CI, 2.2; 95% CI, 1.07 to 4.48). Patients with stage IVA were 20.95 times more likely to die than patients with stage IIB (AHR, 20.95; 95% CI, 6.26 to 70.03). Stage IIIA disease had no significant difference in survival compared with stage IIB disease (*P* = .83; AHR, 0.9; 95% CI, 0.5 to 1.8). Patients with intact rectal mucosa were 0.8 times less likely to die compared with patients with breached rectal mucosa. Prolonged waiting time increased the mortality rate of cervical cancer by 2.9 with a 95% CI of 1.07 to 4.5. Treatment type was also significantly associated with survival (*P* < .014). A single shot was associated with decreased survival of a patient (AHR, 4; 95% CI, 1.4 to 11.1), whereas patients who received only palliative chemotherapy were 4.7 times more likely to die than patients who were treated with radical CCRT (AHR, 4.7; 95% CI, 1.175 to 19.1; Appendix Table A1, online only).

## DISCUSSION

This study makes a significant contribution to the literature regarding cancer control in low-income countries. We analyzed the impact of long waiting times on the progression of cervical cancer in a referral hospital in Ethiopia. Our findings provide evidence of the importance of improving the availability of RT in Ethiopia and similar countries. Furthermore, this research also sheds light on the need for strategies to reduce waiting times for RT.

With approximately 50% of all patients with cancer recommended to receive radiation therapy during their disease course,^11,12^ RT can be used to treat cancers of all stages and types and is often used in combination with other treatments, such as chemotherapy and surgery.^13^ RT can be used as a curative treatment, contributing to 40% of curative treatments for cancer.^12^ It is also used to prevent cancer recurrence or to reduce the symptoms of advanced cancer.^14^

There is a clear disparity in the access and outcomes of RT services between low-income countries, middle-income countries, and high-income countries.^15,16^ The actual coverage of the needs for RT ranges from 34% in Africa to over 92% in Europe to about double the needs in North America.^17^ With only 10% of the world's RT machines located in low-income countries, these countries are particularly disadvantaged owing to the lack of resources and infrastructure for RT services.^18^ As a result, patients with cancer in these countries often have to travel long distances to access RT services, have to wait long times to get service, and often have to pay out-of-pocket for the services. Africa specifically faces a substantial challenge, as in a vast proportion of the continent, RT has to be built up almost from scratch, including the related investments in human and capital resources and the subsequent challenge of maintaining operational capability.^17^ As of March 2020, only 28 (52%) of 54 African countries had access to external beam RT, 21 (39%) had brachytherapy capacity, and no country had the capacity to match the estimated treatment need.^19^ Our study demonstrates that these disparities in access directly affect patients with cancer in Ethiopia, particularly women with cervical cancer who often have to wait long periods for RT services because of the lack of RT machines and resources in the country.^19^ This lack of access to RT results in a long waiting time to receive treatment and poorer patient outcomes.

The oncology department at the oncology center in Ethiopia follows a first come, first served principle because of the high patient volume and ethical concerns, regardless of the radiosensitivity or stage of the disease. This often leads to disease progression, particularly in the case of nonmetastatic cervical cancer, which has a high cure rate with RT. In some low-income countries, chemotherapy is administered to patients with cervical cancer as an adaptive approach to manage the long waiting times for RT.^20^ There is no strong evidence to support this approach. Neoadjuvant chemotherapy followed by surgery has also been used in areas where RT is unavailable.^21^ Data from this area, however, did not show an improvement in survival when compared with surgery alone for early- or advanced-stage cervical cancer.^21,22^ In Ethiopia, patients with cervical cancer are treated with chemotherapy only if they develop symptomatic metastases. Every patient must wait for RT treatment. This study highlighted the impact of this approach.

In the present study, the median waiting time from the diagnosis to the call for CCRT was 477 days. At presentation, almost half of the patients were diagnosed with stage IIB disease. However, approximately 83% progressed to higher stages, indicating worsening conditions.

We compared our findings for waiting time of patients with cervical cancer to receive RT, which was 477 days from diagnosis to treatment, with similar studies conducted in Brazil, Canada, South Africa, and the United Kingdom.^6-8,23^ The median waiting time for patients in Johannesburg, South Africa, in Brazil, and in Ontario, Canada, was 108 days, 41 days, and 27.2 days, respectively.^6-8^ In the United Kingdom, the median waiting time was 14 days in 1996, 18 days in 1998, and 35 days in 2001.^23^ These studies showed significantly lower waiting times than our findings.

In tumor biology, it has been shown that for fast-growing tumors, a delay of 1-2 months can have a significant adverse effect on the outcome, thus minimizing delays from diagnosis to treatment.^24^ At some point in the evolution of every case, the probability of local control decreases sharply over a relatively short period. The maximum rate of decrease in the probability of local control occurs at a 37% local control level when it reaches 25.5% per tumor doubling time.^25^ Cervical cancer is a rapidly proliferating tumor.^24^

In this study, it was found that disease progression occurred after a median waiting period of 51 days. This is comparable with a study conducted in Johannesburg, where tumor progression was noted between 40 and 65 days, with a median progression time of 55 days.^7^ A retrospective analysis of all patients with cervical cancer treated with radical RT between 1990 and 2001 at the Ottawa Regional Cancer Center revealed that longer RT waiting times were associated with decreased survival outcomes in patients treated radically for cervical cancer.^26^ Similarly, our study found that 59 patients (51.30%) had died and the tumor had progressed to a more advanced stage in those who were alive. These findings were not observed in studies at the previously mentioned hospitals, as waiting times were shorter than those in our study.^6-8,23,26^ The percentage of stage II disease decreased from 46.1% to 7.8%, whereas the percentage of stage IVA increased from 17.5% to 26.1%. As a result, a majority of stage II disease cases were only given palliative treatment because of the extended wait time for RT. The intent of treatment changed from curative to palliative in 80 patients (69.6%) because of disease progression. Twenty-five (21.5%) patients did not receive any kind of RT, mostly because they either died while waiting or were unable to come because of their deteriorated clinical condition. Upstaging and changes in the intent of treatment translated into poorer prognosis and decreased survival, as observed in the Kaplan-Meier graphs. Stage and long waiting times have been reported to be significant prognostic factors in patients with cervical cancer waiting for RT.^27^ No association was identified between comorbidities including HIV status and the outcome of patients with cervical cancer in both bivariate and multivariable logistic regression analyses.

These findings highlight the importance of increasing access to RT services in lower-income nations to reduce wait times and provide better cancer care. Potential solutions to increase the availability of RT services in low-income countries include improving infrastructure and access to RT equipment, training and educating health care professionals in the use of RT, and establishing collaborations between health care professionals, government agencies, nonprofit organizations, and donors.^19^ Working together, these groups can help provide the necessary RT services to those in need, significantly improving the outcome of cancer treatment in low-income countries. Furthermore, the national health agency should explore the role of public-private partnership in aiding the scaling up of RT services.

Patients booked for RT did not receive regular follow-up, creating challenges in assessing the precise timing of changes in the natural history of the disease. Difficulties in contacting and tracing the status of some patients at the end of follow-up because of phone communication issues posed a limitation, potentially underestimating the number of deaths. Access to advanced imaging such as MRI is also limited, making it difficult to stage and make decisions on treatment.

In conclusion, the waiting time to receive RT at a referral center in Ethiopia is very long. This results in disease progression, change in treatment intent from curative to palliative care, and increased mortality. We recommend urgent actions to increase access to RT to reduce waiting time and improve the survival of patients with cervical cancer. We also recommend to prioritize early-stage and radiosensitive cancer types, use hypofractionated regimens, incentivize professionals to work nights and weekends, and create an efficient RT management system to ensure effective and efficient use of resources and prioritize patient's needs.

## Figures and Tables

**TABLE A1:**
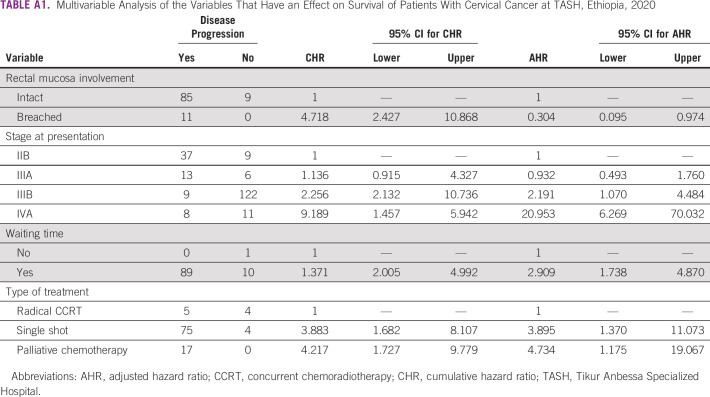
Multivariable Analysis of the Variables That Have an Effect on Survival of Patients With Cervical Cancer at TASH, Ethiopia, 2020

## Data Availability

The data used for this research will be available upon request.
